# Avoiding food avoidance in patients with inflammatory bowel disease

**DOI:** 10.1002/ueg2.12393

**Published:** 2023-04-29

**Authors:** Lihi Godny, Iris Dotan

**Affiliations:** ^1^ Division of Gastroenterology Rabin Medical Center Petah‐Tikva Israel; ^2^ Nutrition Unit Rabin Medical Center Petah‐Tikva Israel; ^3^ Sackler Faculty of Medicine Tel Aviv University Tel Aviv Israel

**Keywords:** diet, dietitian, IBD, quality of life, restrictive eating

Food avoidance is prevalent among patients with inflammatory bowel diseases (IBD). Bonsack et al. recently reported high rates of partial (62%) or total (36%) exclusion of food groups among patients with IBD attending an IBD nutrition clinic in France. Common excluded food groups included raw vegetables and fruits, legumes, and dairy products. Active disease and the use of small molecules or investigational drugs were associated with food avoidance.^1^ This study highlights the emerging and highly prevalent phenomenon of food avoidance in patients with IBD. A recent review examining dietary behaviors in patients with IBD indicated a high prevalence of food avoidance (28%–89%), as well as restrictive dietary behavior (41%–93%).^2^

Patients with IBD avoid foods for various reasons, including

Associating certain foods with GI symptoms including diarrhea, abdominal pain, bloating, and cramps.^3^
Receiving non‐evidence‐based information from alternative therapists, the Internet and social media, leading to unnecessary food exclusion.
(Over) adhering to recommended exclusion diets such as exclusive enteral nutrition (EEN); exclusion diets for active Crohn's disease; the low fermentable oligo, di, mono saccharides and polyols (FODMAP) diet for functional components of quiescent disease; low fiber diet in stenosis and stricturing disease. Without dietitian guidance and long‐term follow‐up, these can lead to unnecessary food avoidance.

Whatever the reason for food avoidance, the outcomes may be deleterious. Specifically:

1. Malnutrition and nutritional deficiencies, which may exist in patients with IBD even without food avoidance. Emerging reports suggest concerning prevalence of vitamin C deficiency and scurvy in patients with IBD,^4^ in addition to common deficiencies like folic acid, B12, calcium and iron, reflecting the extensive restrictions that patients adopt.
2. Microbial alterations reduced microbial diversity and depletion of fiber degrading bacteria resulting from avoidance of fiber containing foods or certain carbohydrates. EEN and low FODMAP diet, despite their favorable clinical effects, induce microbial alterations that further deviate from a healthy microbial composition.^5^, ^6^, ^7^
3. Lower food‐related quality of life (FRQoL) was reported by patients with IBD on restrictive diets.^8^ Impaired FRQoL is associated with lower intake of fiber, calcium, phosphorus and magnesium.^9^ Patients with IBD derive less pleasure from family celebrations and religious occasions because of food restrictions related to the fear of relapse.^10^ FRQoL was noticed to be lower in children with CD compared with their healthy siblings.^11^

Avoiding certain foods can have more harmful effects during specific stages of life, such as deleyed growth in children or can be associated with adverse pregnancy outcomes, and should not be encouraged.^12^

Avoidant restrictive food intake disorder (ARFID) was reported in IBD, with 17% of patients having a positive ARFID score, that correlated with the risk of malnutrition.^13^ While food avoidance is more common in active disease, as was shown by Bonsack, a significant portion of patients report food avoidance during remission phases as well.

Gastroenterologists and IBD nurses can address food avoidance by asking their patients if they avoid certain foods in their diet and if so, refer them to the dietitian for further assessment.

Gastroenterologists should be aware that dietary therapies involving restrictions may lead to side effects like long‐term food avoidance and therefore refer these patients to a dietitian, for further evaluation and shared decisions regarding best approach.

IBD dietitians play a crucial role in preventing and managing food avoidance. They should assess for food avoidance, provide evidence‐based dietary information and communicate the consequences of nutritional inadequacy. Promoting a healthy and diverse diet is essential, while unnecessary and long‐term dietary restrictions should be avoided. Dietitians can recommend changing fiber composition, food texture and preparation, and dietary behaviors to help diversify patients' diets. They should also identify patients in which caution should be used with exclusion diets and ensure that an exclusion diet is followed by a reintroduction phase. Preventing nutritional deficiencies is important, and dietary supplements should be used when needed. Finally, referring patients to psychological support when needed can also be helpful. In the pediatric setting, dietary restrictions can be encouraged by parents of patients with IBD and it is crucial to assess the child's needs and preferences.

**FIGURE 1:**
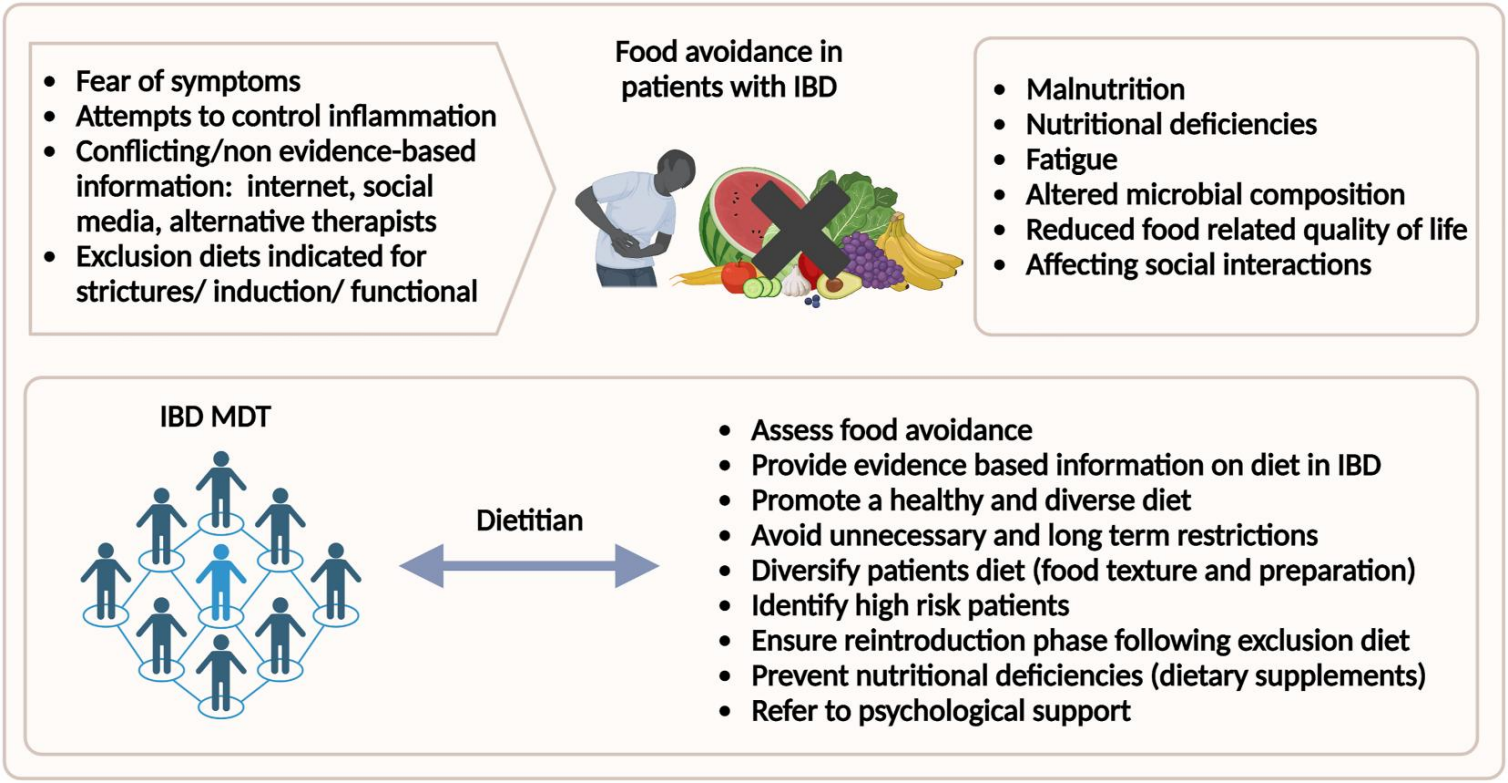
Addressing food avoidance in patients with IBD‐causes, consequences, and strategies to address food avoidance in patients with IBD. IBD, inflammatory bowel disease; MDT, multidisciplinary team. Created in BioRender.com.

Although there is a role for dietary restrictions in several indications, they should be time limited and further development of maintenance strategies that promote healthy and diverse diets is needed. Nutritional education on how to incorporate fiber containing foods in an adapted way to patients with IBD might help in diversifying patients' diet.^14^, ^15^ Multidisciplinary teams that include a dietitian with expertise in IBD, are ideal settings to incorporate dietary therapies and address food avoidance in IBD (Figure 1).
